# Psychometric assessment of the Chinese version of the brief illness perception questionnaire in breast cancer survivors

**DOI:** 10.1371/journal.pone.0174093

**Published:** 2017-03-20

**Authors:** Na Zhang, Richard Fielding, Inda Soong, Karen K. K. Chan, Conrad Lee, Alice Ng, Wing Kin Sze, Janice Tsang, Victor Lee, Wendy Wing Tak Lam

**Affiliations:** 1 Centre for Psycho-Oncology Research & Training, Division of Behavioural Sciences, School of Public Health, The University of Hong Kong, Hong Kong Special Administrative Region, People’s Republic of China; 2 Department of Clinical Oncology, Pamela Youde Nethersole Eastern Hospital, Hong Kong Special Administrative Region, People’s Republic of China; 3 Department of Obstetrics & Gynecology, The University of Hong Kong, Hong Kong Special Administrative Region, People’s Republic of China; 4 Department of Clinical Oncology, Princess Margaret Hospital, Hong Kong Special Administrative Region, People’s Republic of China; 5 Department of Clinical Oncology, Tuen Mun Hospital, Hong Kong Special Administrative Region, People’s Republic of China; 6 Department of Clinical Oncology, The University of Hong Kong, Hong Kong Special Administrative Region, People’s Republic of China; Iranian Institute for Health Sciences Research, ISLAMIC REPUBLIC OF IRAN

## Abstract

**Objective:**

The eight-item Brief Illness Perception Questionnaire (B-IPQ) supposedly evaluates cognitive and emotional representations of illness. This study examined the validity and reliability of a traditional Chinese version of the B-IPQ in Hong Kong Chinese breast cancer survivors.

**Methods:**

358 Chinese breast cancer survivors who had recently ended their primary treatment completed this B-IPQ Chinese version. Confirmatory factor analysis (CFA) tested the factor structure. The internal consistency, construct, predictive and convergent validities of the scale were assessed.

**Results:**

CFA revealed that the original three-factor (cognitive-emotional representations and illness comprehensibility) structure of the B-IPQ poorly fitted our sample. After deleting one item measuring illness coherence, seven-item gave an optimal two-factor (cognitive-emotional representations) structure for the B-IPQ (B-IPQ-7). Cronbach’s alpha for the two subscales were 0.653 and 0.821, and for the overall seven-item scale of B-IPQ was 0.783. Correlations of illness perception and physical symptom distress, anxiety, depression and known-group comparison between different treatment status suggested acceptable construct validity. The association between baseline illness perception and psychological distress at 3-month follow up supported predictive validity.

**Conclusions:**

B-IPQ-7 appears to be a moderately valid measure of illness perception in cancer population, potentially useful for assessing illness representations in Chinese women with breast cancer.

## Introduction

Individuals constructor elaborate illness representations involving both cognitive and emotional elements when facing a health threat or illness [1]. Several core cognitive elements of illness representations have been proposed: consequences, timeline, control, identity, and causes [2]. *Consequences* reflect anticipated or perceived effects or outcomes of illness on an individual’s life. *Timeline* reflects elements of perceived disease duration. *Identity* captures elements including concrete symptoms manifestations and abstract symptom conception and patient beliefs about the illness characteristics. *Control* reflects patients’ beliefs about the extent that they and/or treatment can control the disease. *Causes* reflect patients’ beliefs about why the illness developed. Additional emotional elements of representations include individual’s *concern* about, and perceived *emotional impact* of their illness. These cognitive and emotional components of illness representations were significantly associated with coping behaviors [3], psychological distress [4], and quality of life [5, 6] in previous studies of cancer patients.

The Illness Perception Questionnaire (IPQ) developed by Weinman *et al*. was the first measure of illness perception, assessing five components of cognitive representations of illness [7]. A revised version, the Illness Perception Questionnaire-Revised (IPQ-R) divided the dimension of control into personal control and treatment control, and extended the original IPQ by adding more items and new subscales to assess cyclical timeline perception and emotional representations, and illness coherence, a dimension considered to reflect meta-cognition which was not part of the original formulation [8]. Although IPQ-R is more comprehensive, at 80-items it is lengthy, increasing both response burden and potentially greater non-response and attrition. The Brief Illness Perception Questionnaire (B-IPQ) is a respondent-friendly version that uses the single item that loads highest on each IPQ-R dimension. The original English version of B-IPQ demonstrated good test-retest reliability, and satisfactory concurrent, predictive and discriminant validity [9].

The B-IPQ has been translated into, and validated in different languages. For instance, the Spanish version of B-IPQ was found to be linguistically and conceptually comparable to the original English version [10]. The validation study of Dutch version confirmed its face and content validity, but reported poor test-retest reliability [11]. Using an Iranian diabetic sample, Bazzazian *et al*. [12] reported a low internal consistency (Cronbach’s alpha = 0.53) of B-IPQ in contrast to a Polish version with more satisfactory internal consistency of α = 0.74 [13]. A Chinese version of the B-IPQ showed acceptable test-retest reliability, internal consistency, and construct and discriminant validity in patients with Coronary Heart Disease [14]. Although the original English and translated versions of the B-IPQ had been validated in patients with various chronic illnesses, cancer has not yet been included. Moreover, most studies on cancer-related illness perception were done in Western population. Since cancer patients show cultural and ethnically-attributable variation in cancer perception [15] to pursue studies of cancer in other ethnicities a culturally-valid B-IPQ is required. Furthermore, existing B-IPQ validation studies primarily assessed only the internal consistency and the construct validity. To our knowledge, no study has tested B-IPQ factorial validity. Conceptually, the eight-item B-IPQ captures three aspects of illness perception: cognitive (5 items) and emotional (2 items) dimensions of illness representations and illness coherence (1 item). There is no consensus in the literature whether B-IPQ scores should be interpreted as eight items on three subscales (cognitive and emotional components of illness representations and illness comprehensibility) [16, 17] or as seven items with two subscales (cognitive and emotional components of representations) [18].

To address this literature gap, we examined the psychometric properties of Chinese version of the B-IPQ in a Hong Kong Chinese sample of breast cancer survivors. Specifically, we evaluated data fit for the eight-item three-factor model and the seven-item two-factor model the latter minus the ‘illness coherence’ item. We also tested the internal consistency, construct and predictive validity of the B-IPQ.

## Methods

### Sample size calculation

There is no consensus on how best to compute *a priori* the sample size used to validate a scale [19]. Since the present study used confirmatory factorial analysis (CFA) and maximum likelihood (ML) as the estimation model, the N:q rule (N: cases; q: number of parameters that require statistical estimates) was chosen to calculate the sample size [20]. In this study, q = 26, and an N:q ration of 10: 1 was specified. Thus, the minimum sample size required for our study is 260. To avoid loss of power due to expected drop-out between baseline and 3-month follow-up we increased the targeted sample by ~one third.

### Sample and settings

Following Ethics Committee approval from the University of Hong Kong and Hospital Authority, and as part of a larger ongoing study of over 1,000 women attending five government-funded oncology outpatient clinics and having a known diagnosis of breast cancer who were consecutively recruited between 2010-2013 the first 383 were enrolled for the present study. The inclusion criteria were age> = 18 years, no longer than 6 months since completion of primary treatment (surgery, cytotoxic chemotherapy and radiotherapy), and Cantonese/Mandarin-speaking. Exclusion criteria were linguistic or intellectual incapacity or age over 85 years. Each woman was approached while awaiting their consultation in the out-patient clinic by one of a team of trained research assistants. Written informed consent was obtained from all participants after explanation of the study. Face-to-face questionnaire-based interview was conducted immediately thereafter. Participants were asked to complete a follow-up interview at 3 months post-baseline.

### Measures

#### Illness perception

Illness perception was assessed by the Chinese version of Brief Illness Perception Questionnaire (B-IPQ) [21]. The B-IPQ includes nine items, covering three aspects: cognitive dimensions of illness representations (cognitive representation), emotional dimensions of illness representations (emotional representation), and illness comprehensibility [9]. Cognitive representation is assessed by five items addressing consequences (Item 1), timeline (Item 2), personal control (Item 3), treatment control (Item 4), and identity (Item 5); emotional aspects of illness representations are assessed by two items addressing concern (Item 6) and emotional impact (Item 8). Illness comprehensibility is assessed by one item: coherence (Item 7). All items are scored on an 11-point Likert scale (range: 0-10), with higher scores indicating more cognitive or emotional illness representations. The casual dimension, which being an open-ended question (Item 9), involves patients identifying their three most important perceived causes of breast cancer. Because of this qualitative nature the Casual dimension of representation was excluded from the present study. The Chinese version of the B-IPQ required approximately 5 minutes to complete.

### Comparative measures

#### Physical symptom distress

Physical symptom distress was measured by the Physical Symptom Distress subscale (PHYS) of the validated Chinese version of the Memorial Symptom Assessment Scale Short-Form (MSAS-SF) [22]. This requires patients to indicate if they had experienced any of the listed symptoms over the past 1 week and, if so, rate the intensity of distress caused by each symptom on a five-point Likert scale ranging from 0 “Not at all” to 4 “Very much”. The Chinese version of MSAS-SF has good psychometrics, including internal consistency (Cronbach's α: 0.84-0.91) [22].

#### Psychological distress

Psychological distress was examined by the 14-item Chinese version of Hospital Anxiety and Depression Scale (HADS) [23], which uses a four-point (0-3) Likert scale to assess responses to two 7-item subscales that assess anxiety (HADS-Anxiety) and depression (HADS-Depression) respectively. The possible range of scores for each subscale is 0-21, with higher scores indicating greater level of anxiety or depression. The Chinese version of both subscales HADS-Anxiety (Cronbach's α = 0.86) and HADS-Depression (Cronbach's α = 0.82) demonstrate good psychometrics [23].

All measures were administered at baseline, except for the HADS-Anxiety and HADS-Depression, which were assessed at both baseline and 3 months post-baseline.

Socio-demographic data including age, marital status, education level, occupation, and family income were also collected during the interview. Medical data were collected from medical records.

### Statistical analysis

Descriptive statistics summarized participants’ socio-demographic and clinical characteristics. To assess the factorial validity of B-IPQ, confirmatory factor analysis (CFA) was performed using Mplus version 6 software. Since the B-IPQ scores can be used as an item total or individually as domain scores to assess illness perception, we compared both hierarchical and correlated models of the B-IPQ to determine which gave the best data fit. We used multiple fit indices to evaluate model-data fit, including Chi-squared statistic, root mean square error of approximation (RMSEA) with 90% confidence interval, standardized root mean square residual (SRMR), comparative fit indices (CFI) and Tucker Lewis Index (TLI). RMSEA values≤ 0.10 with 90% confidence interval, SRMR values ≤0.080, CFI and TLI values ≥ 0.90 suggest acceptable model fit [24]. Exploratory factor analysis (EFA) was conducted if both the eight-item three-factor (cognitive and emotional illness representations and illness comprehensibility) and seven-item two-factor (cognitive and emotional illness representations) model were not supported, followed by CFA to verify the revised factorial structure.

Cronbach’s alpha coefficient was used to examine the scale’s internal consistency with the minimal acceptable alpha values of 0.7 [25, 26]. Construct validity was assessed by correlating B-IPQ scores with physical symptom distress (MSAS-PHYS) and psychological distress (HADS-Anxiety and HADS-Depression) scores. We hypothesized that the B-IPQ would correlate with greater physical symptom distress and psychological distress [27]. All correlation was performed by Pearson’s correlation analysis. We also assessed construct validity using a known-group comparison approach (previously had chemotherapy vs. previously did not require chemotherapy). The need for chemotherapy could indicate earlier vs. later disease status. We hypothesized that patients having had chemotherapy were likely to report more negative illness perception than those without chemotherapy. Student’s t-test was used to test the hypothesis. Predictive validity was assessed using multivariate regression to examine the relationship between baseline illness perception and psychological distress at 3-month follow-up. We hypothesized that patients with more negative illness perception at baseline would report a higher level of psychological distress at 3-month follow-up. Apart from CFA, all data analyses were conducted using Statistical Package for Social Sciences version 20.0 (SPSS, Chicago, IL, USA).

## Result

### Sample characteristics

Of 383 eligible breast cancer survivors approached 358 (93.5%) provided full informed consent and completed all the baseline questionnaires, and 286 (79.9%) finished 3-month follow-up assessment. Socio-demographic and clinical characteristics are summarized in Table 1. Patients’ mean age was 51.4 years (SD = 9.7, range = 24-87). Most participants (70.7%) were married, 75.1% had secondary or above education achievement, and 43% were currently employed. The majority (78.0%) were diagnosed with early stage breast cancer. Average duration of time since diagnosis was 10.9 months (SD = 10.6). Almost all participants had surgery as primary treatment, 78.5% adjuvant chemotherapy, and 84.9% adjuvant radiation therapy.

**Table 1:** Socio-demographic and clinical characteristics of the participants.

Variables	*n* = 358
Age (years) Mean (SD)	51.36 (9.65)
Marital status	
Single	55 (15.4%)
Married/cohabited	253 (70.7%)
Divorced/separated	22 (6.1%)
Widowed	28 (7.8%)
Education level	
No formal/primary	89 (24.9%)
Secondary	196 (54.7%)
Tertiary	73 (20.4%)
Occupation	
Employed	153 (42.7%)
Retired	46 (12.8%)
Housewife	70 (19.6%)
Unemployed	89 (24.9%)
Monthly family income (HK$)*	
< = 10,000	78 (21.8%)
10,001-20,000	88 (24.6%)
20,001-30,000	59 (16.5%)
>30,000	108 (30.2%)
Missing	25 (7.0%)
Stage of disease	
0/I	117 (32.7%)
II	162 (45.3%)
III/IV	75 (20.9%)
Missing	4 (1.1%)
Time since diagnosis(months) Mean (SD)	10.92 (10.56)
Type of surgery	
Breast conserving therapy	151 (42.2%)
Mastectomy	179 (50.0%)
Mastectomy and breast reconstruction	24 (6.7%)
Missing	4 (1.1%)
Had chemotherapy	281 (78.5%)
Had radiation therapy	304 (84.9%)
Currently receiving hormonal therapy	226 (63.1%)

* 1US$ = 7.8 HK$

### Factorial validity

Firstly, hierarchical and correlated CFA models were performed on the sample separately to test the eight-item three-factor (cognitive, emotional representation and illness comprehensibility) fit. Then, further hierarchical and correlated CFA models were run on the same sample to test the seven-item (missing the illness coherence item 7), two-factor(cognitive and emotional representations) formulation fit. The goodness-of-fit indices of all the four models are summarized in Table 2. Both eight-item models failed to meet minimum fit criteria. In contrast, the seven-item two-factor hierarchical and correlated models revealed a good fit to the data (SRMR values ≤0.080, RMSEA values ≤0.10, CFI, and TLI all >0.90; Table 2). Because the seven-item (without item 7) two-factor hierarchical model and correlated model were nested, we used Likelihood Ratio Tests (LRT) to compare chi-squared difference. Results indicated that there was no statistically significant difference between the two models in explaining the data (Δ χ^2^ (1) = 0, *P*˃0.05), therefore, the correlated model was preferred on the basis of model parsimony having more degrees of freedom. Hence, hereafter the subsequent reliability and validity assessment is based on the seven-item two-factor correlated model (B-IPQ-7).

**Table 2:** Goodness-of-fit indices of confirmatory factor analyses of the B-IPQ.

Model	χ^2^	df	*P*-value	CFI	TLI	AIC	SRMR	RMSEA(90% CI)
**Eight-item B-IPQ**								
Three-factor hierarchical model	184.681	19	<0.001	0.767	0.657	12784.150	0.137	0.156 (0.136, 0.177)
Three-factor correlated model	91.547	18	<0.001	0.897	0.839	12875.285	0.072	0.107 (0.086, 0.129)
**Seven-item B-IPQ (without IP7)**								
Two-factor Hierarchical model	50.808	13	<0.001	0.944	0.910	5684.718	0.056	0.090 (0.065, 0.117)
Two-factor correlated model	50.808	14	<0.001	0.945	0.918	5684.718	0.056	0.086 (0.061, 0.112)

B-IPQ, Brief Illness Perception Questionnaire

χ^2^: Chi-Square statistic; CFI: Comparative Fit Index; TLI: Tucker Lewis Index; AIC: Akaike Information Criterion; RMSEA: Root Mean Square Error of Approximation; SRMR: Standardized Root Mean-Square Residual.

### Reliability

Cronbach’s alpha for the overall scale of B-IPQ-7 was 0.783, indicating acceptable internal consistency. While the ‘emotional illness representations’ subscale demonstrated good internal consistency (Cronbach's α = 0.821), the ‘cognitive illness representations’ subscale showed only borderline-fair internal consistency (Cronbach's α = 0.653) but the latter comprised the majority of items. This result is not unexpected given the five cognitive items were developed originally to assess different dimensions, and so they may not “scale” well.

### Construct validity

The two B-IPQ-7 subscale scores demonstrated, as expected, moderate positive correlations with physical symptom distress (correlation coefficients (r) ranged from 0.392 to 0.442), anxiety (r ranged from 0.422 to 0.552) and depression (r ranged from 0.429 to 0.494) scores (Table 3). Student’s t-tests were used to test known-group comparison (chemotherapy vs. no chemotherapy). Patient who had received chemotherapy reported significantly higher scores on the “cognitive illness representations” subscale (*p* = 0.016) (Table 4).

**Table 3:** Correlation matrix for convergent and divergent validity.

	‘Cognitive illness representations’ subscale	‘Emotional illness representations’ subscale
Physical symptom distress at baseline	0.392**	0.415**
Anxiety at baseline	0.422**	0.552**
Depression at baseline	0.429**	0.481**

*P*<0.01**

**Table 4:** Known-groups comparisons of B-IPQ mean scores (S.D.) by chemotherapy.

	Chemotherapy		*P*-value
	Yes	No	
‘Cognitive illness representations’ subscale	21.64 (8.71)	18.88 (8.69)	0.016
‘Emotional illness representations’ subscale	9.16 (5.23)	8.49 (5.21)	0.316

### Predictive validity

After controlling for socio-demographic and clinical characteristics, both baseline cognitive representation subscale and emotional representation subscale predicted higher HADS anxiety (*β* = 0.144, *p*<0.05; *β* = 0.405, *p*<0.001, respectively) and HADS depression (*β* = 0.117, *p*<0.05; *β* = 0.347, *p*<0.001, respectively) scores at 3-month follow up (Table 5).

**Table 5:** Standardized betas of multivariate linear regression analyses evaluating the association of B-IPQ-7 with 3-month psychological anxiety and depression.

	Anxiety at 3-month follow up	Depression at 3-month follow up
Age	-	-
Education level		
Primary or below	Referent	Referent
Secondary or above	-	-
Occupation		
Employed	Referent	Referent
Not employed	-	-
Total monthly household income (HK$)		
Below 10,000	Referent	Referent
10,001-20,000	-	-
20,001-30,000	-	-
More than 30,000	-	-
Time since first diagnosis	-	-
Family history	0.115*	0.117*
Surgery	-	-
Chemotherapy	-	-
Radiation therapy	-	-
‘Cognitive illness representations’ subscale	0.144*	0.117 *
‘Emotional illness representations’ subscale	0.405***	0.347***
*R*^*2*^	*0*.*268*	*0*.*242*

*P<0.05

***P<0.001

Dash sign (-) means variables that were excluded by the stepwise multivariate linear regression model.

The italic values are R^2^.

Variables were excluded and retained with p-values (entry) = 0.05 and (removal) = 0.1.

## Discussion

This study assessed the factorial validity, construct validity, and reliability of the Chinese version of the B-IPQ in Hong Kong Chinese breast cancer survivors. Confirmatory factor analysis revealed that the eight-item, three-factor model did not show adequate fit to the data of our sample. In contrast, the seven-item two-factor model showed acceptable fit to the data. This is consistent with Leventhal’s common sense model in which illness perception involves both cognitive and emotional dimensions of illness representations. The deleted item 7 (illness coherence) asked “how well do you feel you understand your illness?”, measuring to what extent individuals’ illness perception reflected a logical and consistent understanding of their illness [8]. This “thoughts about your thinking” is termed meta-cognition [28]. Therefore, it was not surprising that the seven-item two-factor model, omitting this meta-cognition item 7, showed better data fit than did the eight-item, three-factor model. Furthermore, data indicated that the two-factor correlated model was the more parsimonious model in explaining the data. Consequently, ‘cognitive illness representations’ subscale and ‘emotional illness representations’ subscale were confirmed, and two composite scores of the seven-item B-IPQ appear to be most applicable.

The Chinese version of the two-factor B-IPQ-7 showed good internal consistency for both of the overall scale, as well as the ‘emotional illness representations’ subscale. The ‘cognitive illness representations’ subscale, however, demonstrated only marginally acceptable internal consistency. Post hoc examination of the data showed item 3 (personal control) had the lowest item-total correlation (*r* = 0.290) but deleting this item only marginally increased the ‘cognitive illness representations’ subscale Cronbach’s alpha from 0.653 to 0.659. However, to be consistent with the original theoretical model item 3 was retained. The low alpha suggests that scalability may not be particularly reliable for the cognition items. Possible, the instrument is too “brief” and might benefit from the addition of one more item each to the cognitive dimensions. The Chinese version of B-IPQ-7 showed good construct validity indicated by positive correlations with the measures of physical symptom distress, anxiety and depression.

The Chinese version of the B-IPQ-7 demonstrated expected patterns for known-group comparison in terms of treatment status, supporting its clinical validity. Our findings showed that women who had previously received chemotherapy reported greater cognitive illness representations. The Chinese version of B-IPQ-7 also showed good predictive validity in our sample of breast cancer survivors. Adjusting for socio-demographic and clinical characteristics, baseline cognitive illness representations and emotional illness representations subscales significantly predicted anxiety and distress at 3-month follow up.

In summary, our study suggested that the seven-item, two-factor (cognitive and emotional illness representations) Chinese version of B-IPQ (B-IPQ-7) was the best fitting measurement model in Chinese breast cancer survivors, therefore, its use in future studies of two composite scores is supported. This two-factor Chinese version of the B-IPQ-7 demonstrated acceptable validity and reliability for assessing illness perceptions of breast cancer. While a strength of the present study was the adoption of a longitudinal design, allowing assessment of predictive validity, our study sample is limited to Hong Kong Chinese women diagnosed with breast cancer. Future studies should evaluate the psychometric properties of the Chinese version of B-IPQ-7 for use among Chinese patients with other cancer types. Moreover, test-rest reliability was not assessed in the present study. Consideration should be given to doubling the number of items to improve scalability.

## Supporting information

S1 Dataset S1 Dataset for the manuscript. (SAV)

## References

[1] Leventhal H, Nerenz D, Steele D. Illness representations and coping with health threats In: Rachman S, editor. Handbook of Psychology and Health. 4 Hillside, New Jersey: Erlbaum; 1984 p. 219–52.

[2] Diefenbach MA, Leventhal H. The common-sense model of illness representation: Theoretical and practical considerations. J Soc Distress Homel. 1996;5(1):11–38.

[3] Hopman P, Rijken M. Illness perceptions of cancer patients: relationships with illness characteristics and coping. Psycho-Oncology. 2015;24(1):11–8. 10.1002/pon.3591 24891136

[4] Gray NM, Hall SJ, Browne S, Johnston M, Lee AJ, Macleod U, et al Predictors of anxiety and depression in people with colorectal cancer. Supportive Care in Cancer. 2014;22(2):307–14. 10.1007/s00520-013-1963-8 24077745

[5] Mickeviciene A, Vanagas G, Jievaltas M, Ulys A. Does Illness Perception Explain Quality of Life of Patients With Prostate Cancer? Med Lith. 2013;49(5):235–41. 24247920

[6] Ashley L, Marti J, Jones H, Velikova G, Wright P. Illness perceptions within 6months of cancer diagnosis are an independent prospective predictor of health-related quality of life 15months post-diagnosis. Psycho-Oncology. 2015;24(11):1463–70. 10.1002/pon.3812 25946704

[7] Weinman J, Petrie KJ, MossMorris R, Horne R. The illness perception questionnaire: A new method for assessing the cognitive representation of illness. Psychology & Health. 1996;11(3):431–45.

[8] Moss-Morris R, Weinman J, Petrie K, Horne R, Cameron L, Buick D. The revised illness perception questionnaire (IPQ-R). Psychology and health. 2002;17(1):1–16.

[9] Broadbent E, Petrie KJ, Main J, Weinman J. The Brief Illness Perception Questionnaire. J Psychosom Res. 2006;60(6):631–7. 10.1016/j.jpsychores.2005.10.020 16731240

[10] Pacheco-Huergo V, Viladrich C, Pujol-Ribera E, Cabezas-Pena C, Nunez M, Roura-Olmeda P, et al Perception in chronic illnesses: linguistic validation of the revised Illness Perception Questionnaire and the Brief Illness Perception Questionnaire for a Spanish population. Aten Prim. 2012;44(5):280–7. 10.1016/j.aprim.2010.11.022 PMC7025183 21955598

[11] de Raaij EJ, Schroder C, Maissan FJ, Pool JJ, Wittink H. Cross-cultural adaptation and measurement properties of the Brief Illness Perception Questionnaire-Dutch Language Version. Manual therapy. 2012;17(4):330–5. 10.1016/j.math.2012.03.001 22483222

[12] Bazzazian S, Besharat MA. An explanatory model of adjustment to type I diabetes based on attachment, coping, and self-regulation theories. Psychology Health & Medicine. 2012;17(1):47–58. 10.1080/13548506.2011.575168 21678193

[13] Nowicka-Sauer K, Banaszkiewicz D, Staskiewicz I, Kopczynski P, Hajduk A, Czuszynska Z, et al Illness perception in Polish patients with chronic diseases: Psychometric properties of the Brief Illness Perception Questionnaire. Journal of health psychology. 2015. 10.1177/1359105314565826 25589086

[14] Lin YP, Chiu KM, Wang TJ. Reliability and validity of the Chinese version of the brief illness perception questionnaire for patients with coronary heart disease. Journal of the Oriental Institute of Technology. 2011;31:147–57.

[15] Dein S. Explanatory models of and attitudes towards cancer in different cultures. Lancet Oncology. 2004;5(2):119–24. 10.1016/S1470-2045(04)01386-5 14761816

[16] Sampaio R, Graca Pereira M, Winck JC. Obstructive sleep apnea representations, self-efficacy and family coping regarding APAP adherence: a longitudinal study. Psychology, health & medicine. 2014;19(1):59–69. 10.1080/13548506.2013.774430 23484461

[17] Thong MS, Kaptein AA, Vissers PA, Vreugdenhil G, van de Poll-Franse LV. Illness perceptions are associated with mortality among 1552 colorectal cancer survivors: a study from the population-based PROFILES registry. J Cancer Surviv. 2016. 10.1007/s11764-016-0536-5 PMC5018027 26995005

[18] Rizou I, De Gucht V, Papavasiliou A, Maes S. The contribution of illness perceptions to fatigue and sleep problems in youngsters with epilepsy. European journal of paediatric neurology: EJPN: official journal of the European Paediatric Neurology Society. 2016;20(1):93–9. 26497901 10.1016/j.ejpn.2015.10.001

[19] Anthoine E, Moret L, Regnault A, Sebille V, Hardouin JB. Sample size used to validate a scale: a review of publications on newly-developed patient reported outcomes measures. Health and quality of life outcomes. 2014;12:176 PubMed Central PMCID: PMCPMC4275948. 10.1186/s12955-014-0176-2 25492701 PMC4275948

[20] Kline RB. Principles and practice of structural equation modeling: Guilford publications; 2015.

[21] Xue F, Lin Y. Brief illness perception questionnaire: Chinese version [cited 2010 01/08]. Available from: http://www.uib.no/ipq/pdf/B-IPQ-Chinese.pdf.

[22] Lam WWT, Law CC, Fu YT, Wong KH, Chang VT, Fielding R. New Insights in Symptom Assessment: The Chinese Versions of the Memorial Symptom Assessment Scale Short Form (MSAS-SF) and the Condensed MSAS (CMSAS). J Pain Symptom Manag. 2008;36(6):584–95. 10.1016/j.jpainsymman.2007.12.008 18434076

[23] Leung CM, Wing YK, Kwong PK, Lo A, Shum K. Validation of the Chinese-Cantonese version of the Hospital Anxiety and Depression Scale and comparison with the Hamilton Rating Scale of Depression. Acta Psychiat Scand. 1999;100(6):456–61. 10626925 10.1111/j.1600-0447.1999.tb10897.x

[24] Hair J, Black W, Babin B, Anderson R. Multivariate data analysis: Pearson new international edition. Upper Saddle River: Pearson; 2013.

[25] Cronbach LJ. Coefficient alpha and the internal structure of tests. psychometrika. 1951;16(3):297–334.

[26] Bland JM, Altman DG. Cronbach's alpha. Bmj. 1997;314(7080):572 PubMed Central PMCID: PMC2126061. 9055718 10.1136/bmj.314.7080.572 PMC2126061

[27] Zhang N, Fielding R, Soong I, Chan KKK, Tsang J, Lee V, et al Illness perceptions among cancer survivors. Supportive Care in Cancer. 2016;24(3):1295–304. 10.1007/s00520-015-2914-3 26314704

[28] Metcalfe JE, Shimamura AP. Metacognition: Knowing about knowing: The MIT Press; 1994.

